# Emerging K1 serotype *Klebsiella pneumoniae* primary liver abscess: three cases presenting to a single university hospital in Norway

**DOI:** 10.1002/ccr3.77

**Published:** 2014-05-25

**Authors:** Kristoffer Holmås, Aasmund Fostervold, Steen Gustav Stahlhut, Carsten Struve, Jan Cato Holter

**Affiliations:** 1 Department of Infectious Diseases, Akershus University HospitalAkershus, Norway; 2 Faculty of Medicine, University of OsloOslo, Norway; 3 Department of Microbiology, Akershus University HospitalAkershus, Norway; 4 Department of Microbiology and Infection Control, Statens Serum InstitutCopenhagen, Denmark; 5 WHO Collaborating Centre for Reference and Research on Escherichia and Klebsiella, Statens Serum InstitutCopenhagen, Denmark

**Keywords:** Community-acquired infections, K1, Klebsiella pneumoniae, liver abscess

## Abstract

**Key Clinical Message:**

Community-acquired *Klebsiella pneumoniae* primary liver abscess (KLA) has been emerging worldwide over the past two decades and with high incidence in Asia. The presence of specific virulence characteristics is a risk factor for a syndrome with metastatic complications. This report signals an increasing emergence in Northern Europe.

## Introduction

*Klebsiella pneumoniae* primary liver abscess (KLA) has occurred with increasing incidence in Asia since the early 1980s and is now endemic in Taiwan [1]. A specific syndrome with septicemia and metastatic spread to eyes, meninges, or brain may occur and is associated with highly virulent strains of *K. pneumoniae* [2]. KLA is considered a rare disease in Western countries, but in the past decade there has been an increase in the number of reported cases from Europe and North America [3–14]. As of 2013, only one case of this emerging infectious entity (a patient of Asian descent) in Scandinavia has been published [11]. We report the first three cases of KLA in Norway, all of whom were admitted to a single University hospital between 2008 and 2011.

## Case Reports

Major patient characteristics are presented in the Table1.

**Table 1:** Major features of the cases

Characteristics	Case 1	Case 2	Case 3
Gender/age (years)	Female 44	Male 53	Female 40
Origin	Thailand	West Africa	West Africa
Time since visit to native country	8 months	4 years	10 months
Comorbidities	None	Hypertension	Diabetes
Preadmission symptoms	Fever, abdominal pain	Fever, abdominal pain, rigors, dyspnea	Fever, rigors, polydipsia, polyuria, weight loss
Temperature (°C)	38.3	40.2	39.1
WBC count, 10^9^/L	12.4	5.9–14.0	7.1
CRP (mg/L)	320	80–200	150
ALT (U/L)	113	46–170	33
CT scan	Solitary hepatic abscess	Solitary hepatic abscess	Multiple hepatic abscesses
Positive culture(s) for *K. pneumoniae*^1^	Abscess	Blood	Blood
LOS, days	19	12	6

WBC, white blood cell count; CRP, C-reactive protein; ALT, alanine transaminase; AST, aspartate transaminase; *K. pneumoniae*, *Klebsiella pneumoniae*; LOS, length of hospital stay.

1 All isolates were serotype K1, positive for *rmpA*, aerobactin, *kfu*, and *allS*. All strains belonged to MLST 23. None of the patients had extrahepatic manifestations.

### Case 1

A 44-year-old Norwegian female of Thai descent presented with a 6 day history of fever and malaise as well as 3 days with constant abdominal pain in the right upper quadrant. She also reported mild diarrhea, dry cough, and dyspnea.

Her past medical history was unremarkable. She had visited Thailand 8 months earlier. Findings on physical examination included fever of 38.3°C, tachycardia 102/min, tenderness in the right upper quadrant without rebound or guarding. She had no signs of jaundice.

Laboratory findings included a white blood cell count of 12.4 × 10^9^/L, C-reactive protein (CRP) 320 mg/L, slightly elevated total bilirubin 29 *μ*mol/L, alkaline phosphatase 390 U/L, aspartate transaminase (AST) 228 U/L, alanine transaminase (ALT) 113 U/L, albumin 31 g/L, and s-glucose 5.3 mmol/L. Electrolytes and renal function tests were within normal range.

The patient was initially treated empirically with intravenous ciprofloxacin and clindamycin. Ciprofloxacin was rapidly switched to intravenous cefuroxime due to an allergic reaction. Blood cultures were negative.

**Figure 1:**
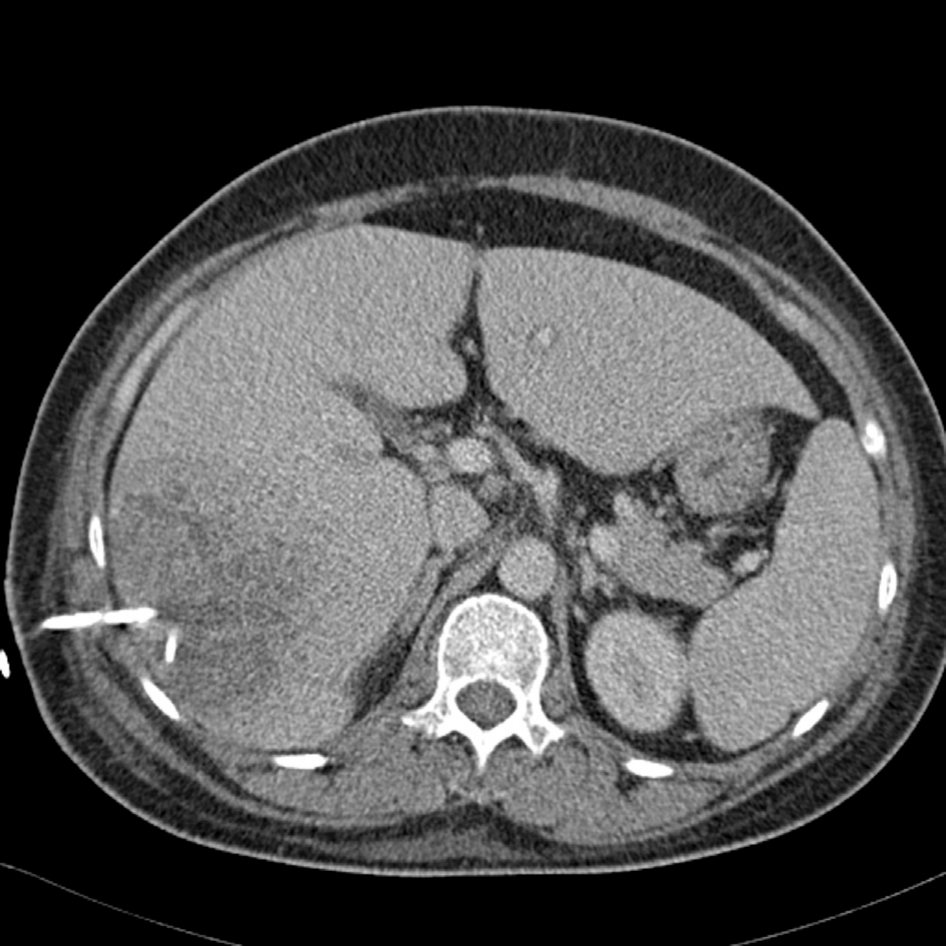
Abdominal CT scan showed a 7.3 × 7.5 cm inhomogeneous abscess in liver segment 6. A therapeutic pigtail catheter was inserted.

A right upper quadrant ultrasound was negative for biliary obstruction, but revealed changes in liver parenchyma. CT scan showed a 7.3 × 7.5 cm inhomogeneous abscess in liver segment 6 (Fig.1). *Entamoeba histolytica* serology was negative. Ultrasound-guided drainage of the abscess yielded purulent fluid, which grew *K. pneumoniae*, sensitive to all antibiotics except ampicillin. A therapeutic pigtail catheter was inserted, and antibiotics were changed to piperacillin–tazobactam. She was discharged after 19 days, and treated with oral ciprofloxacin (i.e., ciprofloxacin 500 mg bid) for another 4 weeks experiencing no allergic reaction. Follow-up CT scan after treatment showed complete abscess resolution.

### Case 2

A 53-year-old male with a past medical history of hypertension developed acute severe malaise with fever and rigors. He also reported thin defecation and intermittent cramp-like abdominal pain accompanied by dyspnea.

The patient was born in West Africa, but had lived many years in Norway. He had not been traveling for the last 4 years. He was in severe distress with a temperature of 40.2°C, but only mild epigastric tenderness was found by thorough physical examination. A urine strip test was positive for leukocytes, protein, blood, and nitritis. Sepsis treatment regimen with parenteral penicillin and gentamicin was initiated. The patient was quickly stabilized.

His white blood cell count was 5.9 × 10^9^/L at admission, rising to 14.0 × 10^9^/L the next morning. He was slightly thrombocytopenic 95 × 10^9^/L and CRP increased rapidly from 80 to 200 mg/L. Liver enzyme values were normal at admission, rising to elevated levels during the first days of hospitalization as he developed signs of jaundice. S-glucose was 6.4 mmol/L.

*Klebsiella pneumoniae* resistant only to ampicillin grew in four of the four blood cultures. Urine cultures were negative. Ultrasound examination showed a nonobstructive biliary system. An abdominal CT scan revealed a 4 × 4 cm intrahepatic abscess in segment 7. Due to the risks associated with an invasive procedure on this localization, it was considered that such intervention could not be justified. Antibiotics were changed to intravenous cefotaxime and metronidazole, which led to rapid clinical improvement. Because percutaneous drainage was not possible, a 6-week course of oral high-dose ciprofloxacin (i.e., ciprofloxacin 750 mg bid) was given. Despite bacteremia, there were no signs of extrahepatic manifestations. A CT scan 10 days after presentation showed initiating abscess resolution. Follow-up CT scan 4 months later showed complete resolution of the abscess.

### Case 3

A 40-year-old female presented with a 6-day history of fever, rigors, malaise, myalgia, anorexia, and weight loss. She also reported polydipsia and polyuria, but no other suspicious symptoms of urinary tract infection or any symptoms suggestive of abdominal involvement. She migrated from West Africa 10 months earlier, and had not been traveling since. Her past medical history was unremarkable.

She was in mild distress with a temperature of 39.1°C and ketonia-smelling breath. A new heart murmur was detected. She had no signs of abdominal tenderness or distension, jaundice, lymphadenopathy, or organomegaly.

Her white blood cell count was 7.1 × 10^9^/L, CRP 150 mg/L, total bilirubin 9 *μ*mol/L, alkaline phosphatase 209 U/L, AST 42 U/L, ALT 33 U/L, s-kalium 4.3 mmol/L, and s-glucose 23.8 mmol/L. Urine strip test showed glucosuria and ketonuria, cultures were negative. Treatment regimen for hyperglycemia was initiated.

Endocarditis was ruled out on the basis of Duke's criteria. *Klebsiella pneumoniae* resistant only to ampicillin grew in one of the four blood cultures and intravenous cefotaxime was initiated. On the third day of hospitalization, she had continued fever and tachycardia. A physical examination now revealed tenderness in the right upper quadrant. The following ultrasound and CT scan demonstrated multiple hepatic abscesses.

Because none of the abscesses were larger than 5 cm, percutaneous drainage was not performed. There were no signs of extrahepatic infectious foci. Antibiotics were continued with combined intravenous cefotaxime and metronidazole.

She responded well, and was further treated as an outpatient with oral high-dose ciprofloxacin for 4 weeks. Imaging and clinical examination 6 weeks after discharge showed no abnormalities (Table1).

## Results/Bacteriology

**Figure 2:**
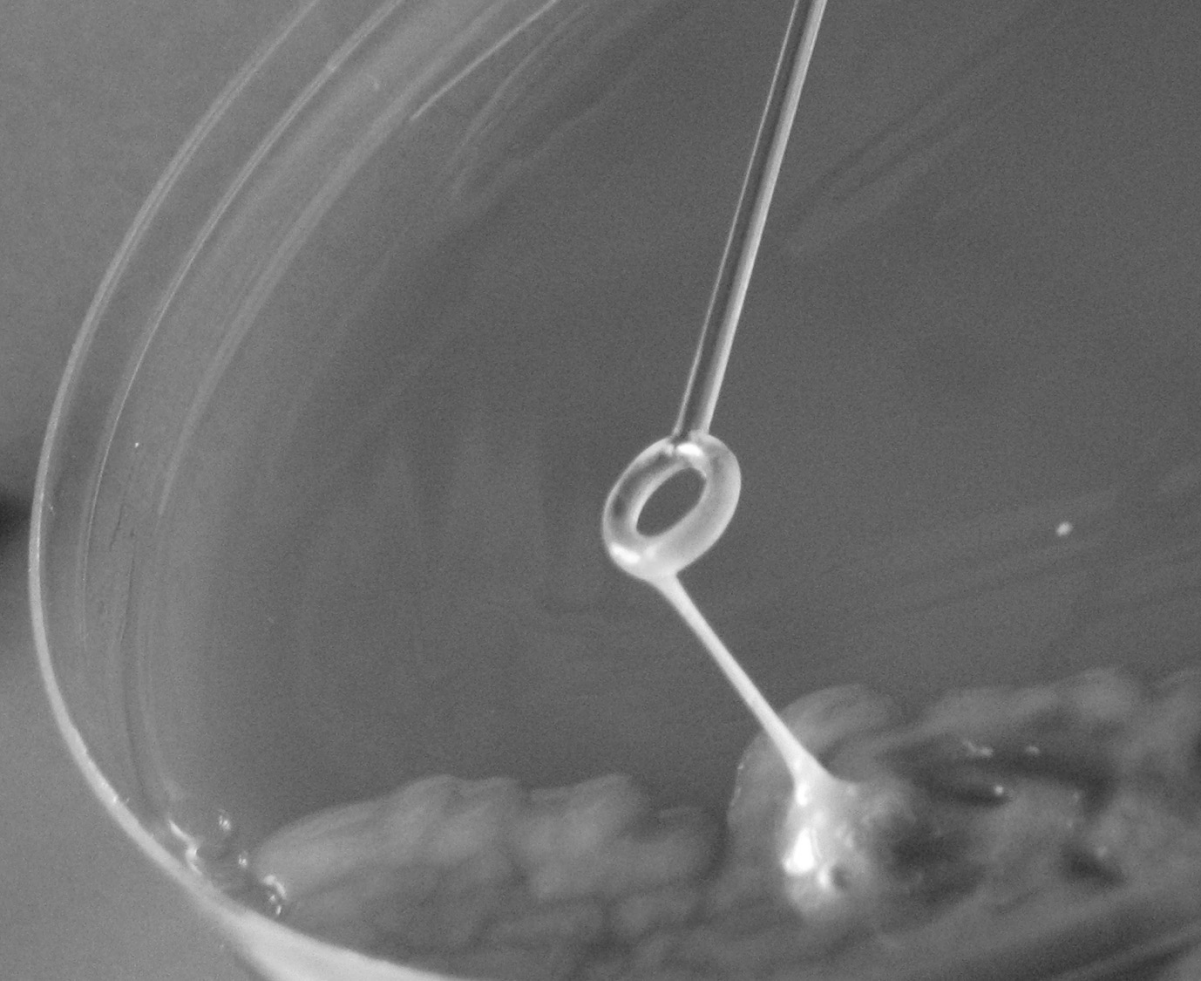
String test. Formation of mucoviscous strings when a loop is passed through a colony.

Virulence characterization was performed retrospectively at the International *Escherichia coli* and *Klebsiella* Reference Centre (WHO), Statens Serum Institut and revealed that the isolates belonged to the K1 serotype which was confirmed by detection of the K1 serotype-specific gene *magA* and expressed the hypermucoviscous phenotype as shown by the formation of mucoviscous strings when a loop was passed through a colony (string test) (Fig.2) [15]. Furthermore, all isolates were positive for all four virulence genes *rmpA*, aerobactin, *kfu*, and *allS* as revealed by polymerase chain reaction using specific primers [2]. Multilocus sequence typing (MLST) revealed that all strains belonged to sequence type (ST) 23.

## Discussion

A distinctive form of community-acquired primary liver abscess with bacteremia and metastatic infection caused by *K. pneumoniae* has been well-known in Taiwan and other Asian countries for two decades [16,17]. Although *E. coli* and polymicrobial infection have been considered the predominant cause of pyogenic liver abscesses in Western countries, *K. pneumoniae* was reported from the United States in 2004 to be increasingly recovered, especially in Asian patients [18]. Primary liver abscess and bacteremia were later connected to the Taiwanese syndrome associated with the highly virulent hypermucoviscous phenotype of capsular serotype K1. The first cases from North America were reported in 2005 and 2007 [3,9]. In recent years, cases from Argentina, South Africa, Belgium, Spain, France, Ireland, and Sweden have been reported, indicating a global dissemination of these strains causing invasive liver abscesses in both Asian and non-Asian patients [4,5,7,11,12,14]. A Korean study provided evidence for clonal dissemination by sequence typing of the serotype K1 *K. pneumoniae* invasive isolates from liver abscesses, showing that these strains were genotypically related and belonged to ST 23 [19]. Recently, both an Argentinian and a French study also reported isolates belonging to ST 23, suggesting its global dissemination [4,12]. Our results showed that the isolates exhibited similar characteristics as the highly virulent *K. pneumoniae* isolates associated with invasive infections in Asia. All isolates belonged to ST 23 supporting the theory of global dissemination. Two of our patients were of West African origin from where there are no published cases of KLA.

The clinical manifestations of KLA are similar to those of pyogenic liver abscesses caused by other etiologies. Although rare, the highly virulent strains of *K. pneumoniae* are more prone to develop bacteremia and metastatic complications that occur in 12–13% of the cases, with eye, meninges, and brain as the most common sites [2,16]. No specific investigations (e.g., CT or ocular examination) were performed to rule out such complications in our patients. However, they were all followed up a long period after hospital discharge without showing any symptoms or signs that would indicate this.

The pathogenetic mechanisms of KLA remain unclear, although several virulence factors of the invasive strains have been described. Capsular serotype K1 of a characteristic hypermucoviscous phenotype has been reported as a major virulence factor of *K. pneumoniae* invasiveness causing the syndrome of primary liver abscess and septic metastatic complications [2,20]. These hypermucoviscous strains also demonstrate high resistance to human serum and phagocytosis killing [15]. Four additional virulence genes, the two plasmid encoded *rmpA* (regulator of mucoid phenotype) and aerobactin (an iron siderophore), as well as two chromosomal encoded virulence genes, *allS* (associated with allatonin metabolism) and *kfu* (encoding an iron uptake system), have also been proven to be important virulence determinants [17,21]. Although rare in other clinical isolates from Western countries, K1 is the most common serotype isolated from patients with KLA [20,22].

Diabetes mellitus or impaired fasting glucose is a major risk factor for primary KLA and the frequency of diabetes among patients with KLA has been reported as high as 78% [20]. A case–control study reported that patients with diabetes mellitus had a statistically significant 2.1-fold increased risk of K1 serotype KLA than of non-*Klebsiella* liver abscess [23]. Poor glycemic control plays a role in impairing neutrophil phagocytosis of these K1 serotype strains which partially can explain these differences [24]. However, it is debated whether diabetes is an independent risk factor for metastatic infection [2,16,17,20]. Of the two bacteremic cases, one was diabetic and the other non-diabetic, thus confirming that these strains are virulent enough to cause severe invasive infection even in a healthy host.

Although the present cases and other reports from Western countries address a global spread of the infection, most patients have been of Asian origin [3,6,8–11,14,18]. Ethnicity and genetic susceptibility may partially explain this pattern. However, earlier colonization in the patients’ home countries has also been suggested as an alternative explanation for the differences in global epidemiology of KLA [13]. A recent Korean study examining fecal carriage of the serotype K1 *K. pneumoniae* ST 23 demonstrated that the isolates from intestinal carriers and liver abscess patients were closely related genotypically, which support this explanation [25]. Alternatively, some patients may have been infected in their social surroundings by someone with a more recent traveling history. This could be the case for one of our patient from West Africa who had not been abroad for 4 years.

Principles of diagnosis are similar to those of other causes to pyogenic liver abscess, including thorough history, clinical examination, radiographic imaging, and laboratory investigation. Serologic tests are useful in such cases. If possible, purulent material from needle aspiration should be Gram stained and cultured for aerobic and anaerobic bacteria. Underlying hepatobiliary- or pancreatic disease must be considered in general. Risk factors for KLA, such as diabetes and geographic origin, must be assessed in particular by the clinician for early diagnosis. This will be guided by bacterial identification and a simple string test can easily detect the hypermucoviscous phenotype for confirmation [15]. Rapid detection of the virulent K1 serotype will be most helpful in diagnosis and treatment to decrease the risk of severe metastatic infections, as well as in epidemiological studies [26].

Community-acquired KLA isolates rarely produce extended-spectrum β-lactamases (ESBL) [27]. In Norway, the prevalence of ESBL-producing *K. pneumoniae* isolates in blood cultures is 2.9% [28].

Treatment of KLA requires parenteral antibiotic therapy combined with percutaneous drainage when possible. Drainage should follow the same guidelines as for pyogenic liver abscesses of other causes [29].

Initial empirical antibiotic treatment should be guided by local bacterial resistance patterns and subsequently be tailored to culture- and antibiotic susceptibility results. Norwegian guidelines for hepatic abscess treatment recommend intravenous piperacillin–tazobactam or ceftriaxone and metronidazole for 10–14 days until clinical improvement, followed by oral ciprofloxacin and metronidazole for a total treatment length of 4–6 weeks. Longer courses of treatment may be warranted for patients requiring subsequent drainage procedures or with persistent radiographic evidence of abscess. In general, treatment should be continued until follow-up CT imaging demonstrates complete or near complete resolution of the abscess cavity.

The prognosis of KLA is good if early and adequate treatment is given. Mortality rates range from 2.8% to 11.3% and relapse rates from 4.4% to 6.5% [2,16,30]. Although the mortality rate is relatively low, metastatic infection may cause considerable optic and neurologic disabilities [2,16].

This report presents the first three published cases of hypermucoviscous serotype K1 *K. pneumoniae* liver abscess in Norway. All cases were presented to a single University hospital, which may signal an increasing emergence in Northern Europe. Awareness by clinicians and public health officials could allow early detection and optimal management of this condition which also affects non-Asians. Further geographic dissemination of hypermucoviscous K1 *K. pneumoniae* strains of ST23 is likely and a rising number of cases from other geographic regions indicate that this is a globally emerging infectious disease.
